# The Study of Randomized Visual Saliency Detection Algorithm

**DOI:** 10.1155/2013/380245

**Published:** 2013-12-09

**Authors:** Yuantao Chen, Weihong Xu, Fangjun Kuang, Shangbing Gao

**Affiliations:** ^1^School of Computer Science and Engineering, Nanjing University of Science and Technology, Nanjing 210094, China; ^2^School of Computer and Communication Engineering, Changsha University of Science and Technology, Changsha 410114, China; ^3^Department of Computer Science and Technology, Hunan Vocational Institute of Safety & Technology, Changsha 410151, China

## Abstract

Image segmentation process for high quality visual saliency map is very dependent on the existing visual saliency metrics. It is mostly only get sketchy effect of saliency map, and roughly based visual saliency map will affect the image segmentation results. The paper had presented the randomized visual saliency detection algorithm. The randomized visual saliency detection method can quickly generate the same size as the original input image and detailed results of the saliency map. The randomized saliency detection method can be applied to real-time requirements for image content-based scaling saliency results map. The randomization method for fast randomized video saliency area detection, the algorithm only requires a small amount of memory space can be detected detailed oriented visual saliency map, the presented results are shown that the method of visual saliency map used in image after the segmentation process can be an ideal segmentation results.

## 1. Introduction

The researchers in recent years have made a lot of content-based image and video image scaling method [1–7]. These images and video scaling methods are intended by changing the ratio of the image or video and the resolution so that the image or video to the terminal equipment suitable for the target display, and try to save your images and video in the critical content. In these images based on image content and video scaling process, how to quickly detect visual saliency areas is needed to solve the problem.

Now image pixels based point in visual saliency region detection method [8–11] are mostly single pixel of each calculation system significantly, because the large number of pixels will result in an overall computation huge. Some methods are even also building high-dimensional vector to perform the search tree structure [8], and the method's time complexity and space complexity are much higher than other method. Therefore, many existing regional visual saliency detection method [8, 9] can only detect relatively rough area significant results. The literature [10, 11] of the proposed method is made from the image analysis spectral angle to calculate the input original image on the saliency region. The literature [12] is based on machine learning methods to obtain the input of the original image visual saliency area. These methods can accurately detect the original image smaller target, mainly used in target identification and target tracking. They are proposed for the randomization of visual saliency detection methods with the literature's [8] method is mainly used in image processing of the visual detection and image saliency map area.

According to the literature [8, 13], they are related to outcomes inspiration, and the paper have proposed a novel randomized visual saliency detection algorithm, which is based on high-quality content visual saliency regional approach. In the method, the first application of randomized looking algorithm for rapid detection, the input image belongs pyramid each corresponding roughly saliency region. Secondly, rough visual saliency region meticulous process, and remove as randomized seeking algorithm to generate noise signal, once again the image pyramid of different levels of consolidation for saliency region careful treatment. Finally, for each pixel, adaptive update saliency value, resulting in detailed oriented saliency final result.

The paper has described the randomized visual saliency detection algorithm and it is a randomized algorithm. It requires no additional data structure to construct auxiliary systems saliency region detection related work, and only need to store the original input image and the system output saliency results figure required memory to be able to perform. The randomized algorithms have quickly produced from the original input image size and exactly the same meticulous system visual saliency area graph. The randomized algorithms can be easily performed on the graphics processing unit to achieve even parallel computing. These advantages make the proposed efficient randomized visual saliency region detection method become used in video sequences in real time. The system has generated from the corresponding visual saliency map, thereby improving image content-based video scaling to generate the overall quality of the results.

## 2. Image Visual Saliency Detection

Image processing saliency region detection has been the fields of computer vision problems remain unresolved. At present, significant dependence on specific areas related applications will generate many significant regional differences customized versions with a variety of regions of interest ROI detection algorithm coexist. Existing significant region detection algorithm is mainly focused on looking for the human visual attention first fixed pixel or object. The visual saliency for understanding human has visual attention pixel and related application, such as auto-focus applications. While the rest of the visual saliency detection algorithms are more emphasis than the specific detection of the single object in the image.

According to the literature [8], the earliest of the early original behavior of the human visual system and neural network architecture combining visual attention mechanism put forward a visual saliency system. The literature [8] had proposed the algorithm for multiscale image features that are combined to obtain unitary visual saliency map. Image multiscale features include six brightness feature maps, twenty four orientation maps, twelve colorful characteristics of the map. To be able to quickly detect multiscale features of the original image, the literature's [8] method only cursory level calculated to be roughly characterized map. In fact, the method in literature [8] had generated a rough visual salience effect corresponding region map algorithm and different methods, the proposed method is to generate the degree of detail of the input image size exactly matches the original visual saliency map.

The literature [9] in the context of the proposed content-based visual salience, this algorithm aims to detect the input image can represent a specific area of the scene. Other people had think pixel visual saliency is determined by the pixel area of the center of the image related to block said, because the region reflects the pixel block location of its context. If the center pixel of the relevant image region block *i* is shown as *p*_*i*_ and the other image region in the original image block differences is large. It can be seen image pixel *i* as highly visual saliency pixels.

Definition of *d*_color_(*p*_*i*_, *p*_*j*_) is the result of the image area to the quantized block *p*_*i*_ and *p*_*j*_ on the CIE Lab color space separated by the Euclidean distance, and normalized to correspond to the [0,1]. When *d*_color_(*p*_*i*_, *p*_*j*_) is relatively large in any image area block *p*_*j*_, then the image pixel *i* considered statistically visual saliency.

*d*_position_(*p*_*i*_, *p*_*j*_) is the image area defined the Euclidean distance of block *p*_*i*_ and block *p*_*j*_ between the locations, this corresponds to the normalized distance [0,1]. Based on these two definitions in the literature [8], the measure of two corresponding blocks of an image area no similarity measure between the methods are shown in formula (1). Consider

(1) $$\begin{array}{c} d\left(p_{i},p_{j}\right)=\frac{d_{\text{color}}\left(p_{i},p_{j}\right)}{1+c\times d_{\text{position}}\left(p_{i},p_{j}\right)}. \end{array}$$

The literature [9] had proposed the method considers only the *K* blocks of high similarity image area (if the area of the image block high similarity with the image block *p*_*i*_ are distinct from, then the entire image area in the original image are obviously different block in the image area block *p*_*i*_). Therefore, for each image block *p*_*i*_, the original input image in accordance with formula (1) to find the highest similarity of *K* blocks of the image area, and according to formula (2) to calculate the pixel position *i* visual saliency. Consider

(2) $$\begin{array}{c} S=1-\exp\left\{-\frac{1}{K}\sum_{k=1}^{K}d\left(p_{i},p_{k}\right)\right\}. \end{array}$$

The literature [9] had implemented methods can only detect single width or height is 256 pixels rough saliency map image area. Because each pixel need to be independent saliency calculations, so a very large amount of computation, and in order to Find the fastest most similar block of the image area, you need to construct a sparse grid lookup based on high-dimensional vector tree. Therefore, if the detailed oriented images individually calculated for each pixel of the saliency value, the whole process will be very slow. This idea is based on the Goferman's improvement of the proposed method and others, but not all the pixels in the entire image to find the area of the image most similar block to calculate the pixel saliency value, instead of using a random method all pixels from the image dot image regions extracted 2*K* blocks, and to ensure image regions where *p*_*i*_ of the highest similarity blocks and blocks of the image area, the image area of the other blocks of the *K* discarded. This improved method of extraction as long as 2*K*randomized blocks image regions, but do not create an auxiliary high-dimensional vector lookup tree structure to improve search efficiency. Compared with the conventional variety, this improved method of the time is short, less space.

In the literature [8, 9], the method can effectively detect the input of the original image with the visual saliency image related areas. Because depending on the image on the original image all the pixels directly calculate the corresponding visual saliency map, computation is very large and take up more memory area. So the literature [8, 9] just got a rough diagram of saliency. The proposed method can be constructed directly on the original image detail oriented visual saliency map, the detailed oriented visual saliency map will be in many image processing or video scaling processing and other applications on a variety of application areas.

**Figure 1:**
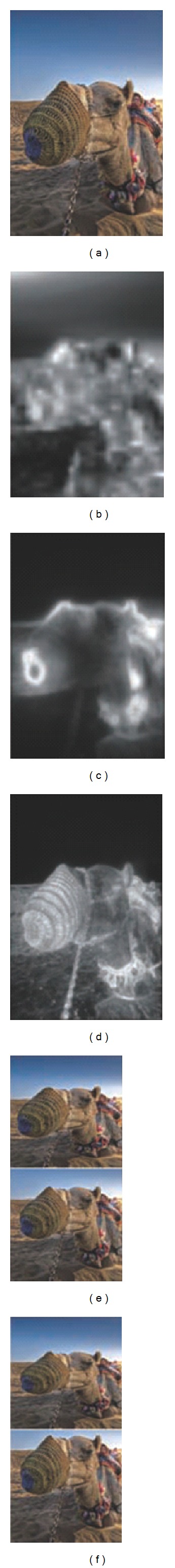
Saliency regional results and graphics scaling results.

Figure 1 can show the detailed use of visual saliency map application Scale-and-Stretch method to perform image scaling relevant scaling results obtained. The experimental results show detailed oriented visual saliency map scaled to produce better results.  Figure 1(a) of the input image size is 476 × 704. According to the literature [8]'s method, Figure 1(b) is shown as visual saliency map.  Figure 1(c) under the literature [9]'s method of visual saliency map.  Figure 1(d) is using a random method for detecting the visual saliency, and Figure 1(e) of the figure has produced by the method according to the visual saliency recovery results of the literature [9].  Figure 1(f) of the picture is shown as the general result of the input image.  Figure 1(f) the following figure is shown as the method according to the random visual saliency map recovery results.

**Figure 2:**
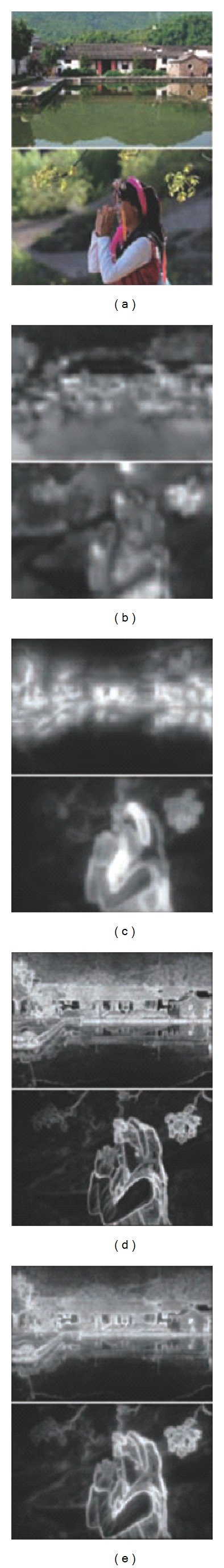
Visual saliency maps of the results.

Figure 2 is shown as the comparison of the paper's method and Itti method, Goferman method, the method can get a high degree of detail of the visual saliency map.  Figure 2(a) is the size of 742 × 495 for the input image. According to Itti method, Figure 2(b) had produced the visual saliency. According to Goferman method, Figure 2(c) had produced the visual saliency.  Figure 2(d) is shown as the randomization method according to this layer for detecting a rough visual saliency.  Figure 2(e) had produced according to the Gaussian image pyramid of multilayer detailed visual saliency map.

## 3. Randomized Visual Saliency Detection Method

The detection method have proposed in the paper is divided into four stages. (1) The first stage is carried out according to the original input image processing, application information at various levels of Gaussian image pyramid to get rough on all levels of the visual saliency map. (2) The second stage is to use the a coarse level of visual saliency detailed diagram of step, the purpose is to remove coarse of the visual saliency map as various image generated randomization noise signal. (3) The third stage through the multilayer careful visual saliency map of the combined Resultant combined with multiscale feature visual saliency map. (4) The fourth stage selectively noise signal for those regions of high integration significantly after the merger to obtain the final results of the visual saliency map. The results for the saliency map modest degree of detail, but if required to achieve a high detection speed, you can use this randomized visual saliency detection algorithm in the first two stages to generate roughly the speed of the visual saliency map. When the degree of detail required and the original input image exactly match the same visual saliency map when fully implemented in four phases may be applied to multiscale feature for image processing, high-quality produce detailed-oriented visual saliency map.

The original image at the two dimensional coordinates of the mapping function *f* : *P* → *S* is used in the process of random saliency map (RSM). It is to be in the original input image coordinates of all pixels in the two-dimensional coordinates defined on the mapping function *f*; *S* represents the normalized execution obtained after the process of visual salience values. Located in the original input image pixel *p* on *P*. *P* corresponds to the original input image in [0, 1] on the visual saliency value of *s*, the mapping function of *f*(*p*) = *s*. The function's values are normalized to [0, 1] within the visual saliency values are stored in the same size and the *P*-dimensional array.

### 3.1. Randomization Visual Saliency Detection

Let *s* = *f*(*p*) be through with multiple pixel *p* in close proximity with the relevant image region blocks for visual saliency value calculation, the specific methods such as formula (3) below. Consider

(3) $$\begin{array}{c} p_{i}=p+\omega \alpha^{i}R_{i}. \end{array}$$

In formula (3), *R*_*i*_ is a random distribution of the variable. Its value is limited to the range of [−1,1]×[−1,1]; *w* is the original input image size 1/2; *α* selection window is the attenuation factor, which is used to the *i* = 1 to *i* = 2*K* images regional blocks until search radius reduced to a single pixel. If *i* < 2*K*, then *i* = 1, the candidate region has been the test until the number of blocks is 2*K*. In this follow-up implementation section, *α* = 0.75.

According to the formula (1) in the candidate region 2*K* blocks of the candidate area is calculated in the block is not the similarity between the *P* value. This algorithm preserves only 2*K* candidate in an image block in 1/2 dissimilarity values smaller blocks of the image area, discard the remaining 1/2 block of the image area. According to the formula (2) image area reserved for the *K* block coordinates the visual saliency *p* value calculation process. In this paper of the experimental, part *K* is 32. *P* in accordance with the pixel selection anywhere approximate 2*K* blocks of candidate image region, the block is similar to the original input image area image *P* in all possible partial region of the candidate region, as this is not exactly of the sample collection process and therefore not entirely sampling will give up to get a sample of the visual saliency map introduced a degree of sampling error.

**Figure 3:**
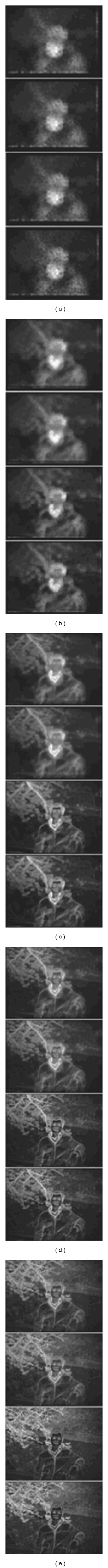
Various stages of saliency results figure.

However, with the number of samples 2*K* gradually becoming large, there certainly will be significantly reduced sampling error.  Figure 3 is the application of the fourth line image. The randomized significant value is calculated after the calculation of the image in the Gaussian pyramid rough layer obtained after the detection of the visual saliency map.

### 3.2. Detailed Visual Saliency Map

As mentioned above, the use of randomization means of testing to get a rough visual saliency map, as well as an insufficient number of samples collected random sample error and other reasons will lead to a lot of noise random exist.

In Figure 3(a) on the fourth line in the resulting image, the expression of the direct access of a rough visual saliency map, this line can be found in the visual saliency map contains a large randomized noise. The random noise is the cause of randomly selected image region 2*K* block execution formula (3) to calculate the noise generated. This after the relevant comparison, using eight neighborhood visual salience values, can be rough for the visual saliency map of meticulous execution process. Eight neighbor method from the pixel coordinates of the point *p* corresponding to the eight directions for selection of neighboring pixels by eight neighbor candidate coordinate method for image region obtained randomly selected candidate block and the domain block image is very different ways. Because *p* coordinates neighborhood image similarity is high, so the eight neighbor coordinate method may make *p* coordinates saliency value be higher than the actual image saliency value smaller; if according to this method to obtain the saliency values normalization process, will lead to the aforementioned resultant rough visual saliency map of the corresponding noise generated. But neighboring coordinates saliency and adjacent to such high similarity, so need for the neighborhood saliency values quite different pixel coordinate positions detailed oriented. We chose detailed oriented neighborhood with eight large differences between visual salience values, because they are not high enough credibility.  Figure 3 on the third line of the image is done by this randomization method for visual saliency in Section 2 of the proceeds of roughly a careful visual saliency map of the corresponding results obtained. The third line in Figure 3 highlights the saliency map detailed map of the visual saliency than a rough map of the visual effect significantly smoother and clearer.

### 3.3. Multilevel Visual Saliency Consolidation Area Map

Delicate area of visual saliency map also cannot achieve complete removal of the noise signal. To multiscale visual saliency feature further into the visual saliency map the final results of which will be adjacent to a rough of visual saliency maps and detailed oriented visual saliency map fusion and aggregated to the visual saliency map of the specific expression in formula (4) shows

(4) $$\begin{array}{c} {\text{RSM}}_{\text{merged}}^{i}=\text{merge}\left({\text{RSM}}_{\text{refined}}^{i},{\text{RSM}}_{\text{refined}}^{i-1}\right). \end{array}$$

In formula (4), RSM_merged_^*i*^ is *i*th layer through the combined saliency map randomized, RSM_refined_^*i*^ is *i*th layer of the randomized after careful visual saliency map.  Figure 3 on the second line of the visual saliency map is detailed through the different levels of visual saliency map obtained after merging relevant results. RSM_refined_^*i*−1^ is a layer closest to the *i* first to *i* − 1 layer of fine visual saliency map. If the size of RSM_refined_^*i*−1^ and RSM_refined_^*i*^ are different, then the merger has made that levels would need to be the size of RSM_refined_^*i*−1^ to RSM_refined_^*i*^ sizes closer.

Try using the weighted algorithm processes, the visual saliency map of the combined method, and the average combined visual saliency map meticulous approach to the visual saliency map of the combined operation. And after the relevant experimental results show that the combined weighted saliency, map method can be significantly higher than the average visual diagram consolidation method better. In the *i*-layer *p* coordinate position, detailed oriented saliency value compared to *S*_(*r*,*p*)_^*i*^, *S*_(*r*,*p*)_^*i*^ which is normalized to [0,1] range. To calculate *i*th layer combined saliency values, the first *i* − 1 layer on a rough visual saliency map will be adjusted to *i*th layer and visual saliency map the same size. Use of meticulous visual salience values expressed in scaled *S*_(*r*,*p*)_^*i*−1^ after the operation of the visual saliency map coordinate position on RSM_refined_^*i*−1^ visual salience values *p*. The adjacent layer of detailed visual saliency value is the result of the merger shown in the following formula. Consider

(5) $$\begin{array}{c} S_{\left(m,p\right)}^{i}=w_{p}^{i}S_{\left(r,p\right)}^{i}+w_{p}^{i-1}S_{\left(r,p\right)}^{i-1}, \end{array}$$

(6) $$\begin{array}{c} w_{p}^{i}+w_{p}^{i-1}=1.0, \end{array}$$

(7) $$\begin{array}{c} \frac{w_{p}^{i}}{w_{p}^{i-1}}=\frac{S_{\left(r,p\right)}^{i}}{S_{\left(r,p\right)}^{i-1}}. \end{array}$$

According to formula (6) and formula (7), in the first layer detailed oriented saliency value *S*_(*r*,*p*)_^*i*^ and *i* − 1 layer detailed oriented visual saliency value *S*_(*r*,*p*)_^*i*−1^ have been identified in the case, you can calculate *w*_*p*_^*i*^, *w*_*p*_^*i*−1^, when *S*_(*r*,*p*)_^*i*−1^ = 0.0, then *S*_(*m*,*p*)_^*i*^ = *S*_(*r*,*p*)_^*i*^. According to formula (5) calculation of the combined fine of saliency values, Figure 3 of the second line saliency map is the result of combined effects of saliency map, which shows through the combined operation of the visual saliency map can be two levels of each saliency multiscale feature aggregated, and the resulting saliency results figure smoother and clearer.

In most cases, a multilevel implementation of the merger proceeds saliency map has been able to achieve the expected results. The visual saliency map algorithm can be suitable for basic image editing application. And Section 3 of the paper some of the same, set at *p* coordinates appear higher saliency value indicates an image with a higher reliability. Therefore, in the most cursory of neighboring hierarchy for visual saliency map updates replace the original merger levels above the saliency map. In Figure 3, the second to the fifth are been updated saliency value of saliency map. In summary, it can be seen from Figure 3, the first line of the fifth saliency map is this randomized saliency detection results obtained optimal saliency map.

## 4. The Experimental Results and Analysis

Using Matlab 2012b simulation development environment for the proposed algorithm of the paper is implemented and simulated in the Microsoft Windows 7 operating system environment to achieve this randomization visual saliency detection algorithms, and uses this randomized saliency detection algorithm based on a lot of the original input image generation results of a large number of saliency map. All this to get results saliency map are in the Pentium R Dual-Core CPU E8400 generated on individual PC. This got a lot of visual saliency results chart shows randomized visual saliency detection methods available in different levels of complexity of the original input image to obtain good results for image detection effect and visual saliency map, but also by the paper randomized visual saliency detection methods produced results visual saliency map and Itti methods, Geforman methods were compared, and the resulting visual saliency map detected more clear and detailed. Figures 5 and 6 shows this method to generate a plurality of randomized visual saliency results map.

It can be seen from Figure 1 that this randomized saliency detection method can produce with the size of the original input image *P* of the same meticulous visual saliency map. It can be detailed oriented visual saliency map of the image content scaling process conducted applications, than Itti method, Goferman obtained by the method saliency map has better image zoom effect. It also describes using detailed oriented visual saliency map to the original input image significant areas of the visual content of the image scaling results more realistic figure.

Figure 2 shows that the use of randomized saliency detection method produces results saliency map method than the application Goferman detection based on image content obtained Itti visual saliency maps and methods of visual saliency map clearer and more detailed.

**Figure 4:**
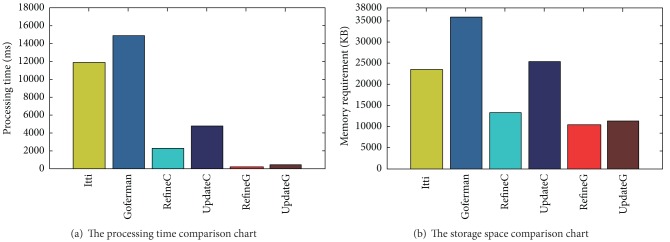
The comparison of randomize methods, Itti, Goferman.

Figure 4 randomization methods and Itti methods, Goferman methods were compared. The Experimental results show that the experimental treatment with a size of 800 × 600 of the original input image, the method of randomization is very relevant stages of processing time significantly lower than Itti method, Goferman methods; and the randomization method if applied to the video card's GPU, execution time is exponentially decreasing number. For the same original input image correlation processing, the system memory usage method comparison results show that the application Goferman highest share memory space requirements, and the application of the randomized saliency detection method memory usage is less than Itti methods and Goferman method, the method of randomization if applied to the video card's GPU, the memory space it occupies will definitely decrease.

**Figure 5:**
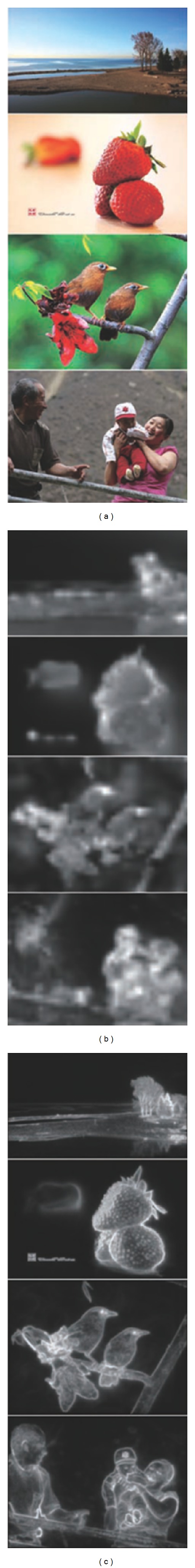
Randomize method and Goferman produces visual saliency results maps: (a) the input image; (b) Goferman produces visual saliency results; (c) randomize method produces visual saliency results.

**Figure 6:**
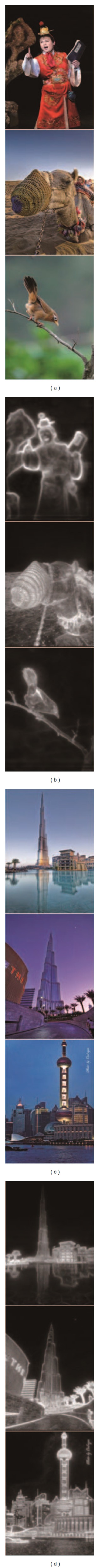
Visual saliency results example: no. 1 and no. 3 are the input image; no. 2 and no. 4 are the application of the randomize method produces visual saliency maps.

Figure 5 shows the randomized saliency detection methods resulting saliency map and Goferman methods of visual saliency map performs the comparison relevant results, the comparison result indicates that the randomization method produces visual saliency map results Figure more meticulous.  Figure 6 shows the randomization method produces significant results other visual diagram instance.

Colorful image segmentation algorithm is currently assessed are generally subjective judgment by the human eye, the author proposed algorithm is applied to image segmentation results with standard libraries Berkeley image segmentation results were compared with the human eye to the right algorithm for qualitative assessment.

**Figure 7:**
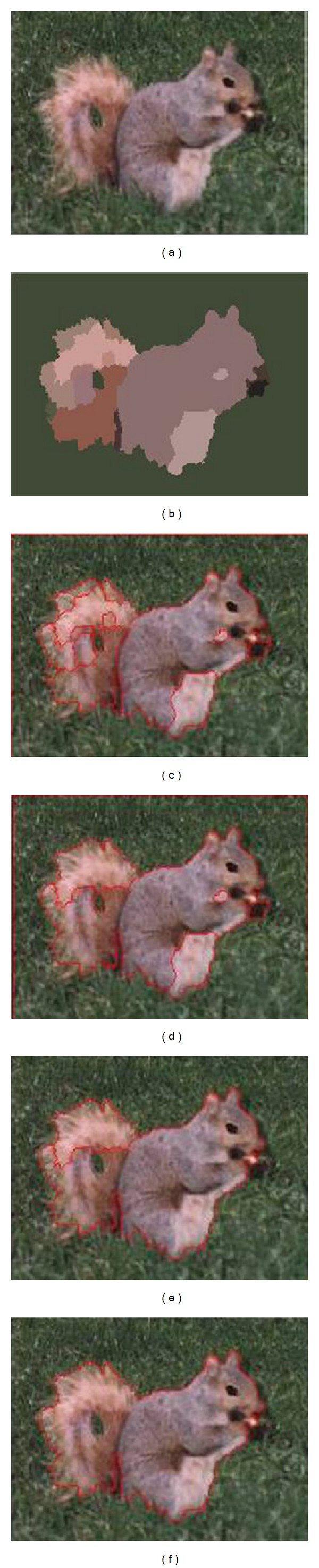
The image segmentation algorithm in the paper.

Figure 7 for the application of this randomized saliency detection algorithm for single image for image segmentation of the specific process; (a) is the original color image, (b) is the first stages of the coarse visual saliency map, (c) is for rough flower visual saliency map stepwise detailed oriented resulting visual saliency map, (d) is a combination of multiscale features of the visual saliency map, (e) is obtained after the final integration of the visual saliency map, and (f) is based on the final visual saliency map image segmentation procedure performed after the final segmentation results obtained.

**Figure 8:**
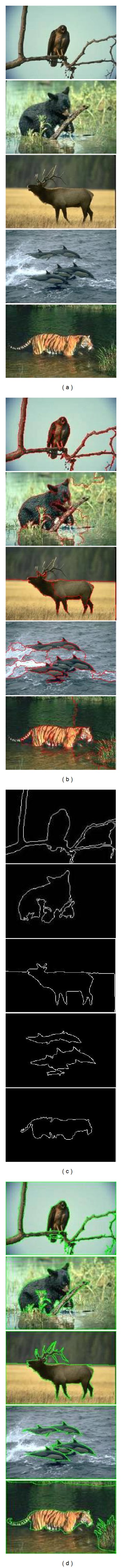
Image segmentation results comparison maps.

Figure 8 are listed separately using the standard algorithm for image library part Berkeley image segmentation results while the application lists the mean shift algorithm as a result of the comparison as well as the human eye horizontal segmentation results, Where Figure 8(a) is the original color image, Figure 8(b) for the application of mean shift algorithm for image segmentation results, Figure 8(c) for this article randomized saliency detection algorithm for image segmentation, and Figure 8(d) the human eye segmentation results. From the segmentation results in Figure 8, it can be seen the use of classical mean shift algorithm in color image detail areas cause over-segmentation, the proposed randomized saliency detection method is according to the saliency map for significant regional location, access to and the human eye split almost consistent image segmentation.

## 5. Conclusions

The paper has presented the randomized visual saliency detection algorithm. The randomized visual saliency detection method can quickly generate the same size as the original input image and detailed results of the saliency map. The randomized visual saliency detection method can be applied to real-time requirements for image content-based scaling saliency results map. The randomization method for fast video randomized significant area detection, this algorithm consists of four phases: the first use of randomization visual saliency detection method first generates roughly oriented visual saliency map. The second stage for generating rough video significant results figure careful treatment, removal of correlated noise signal. The third stage for a rough video saliency map to merge in order to extract multiscale features to obtain a multilevel video significant results figure. The fourth stage for video significant results has enhanced graph merging of the final high-quality video and detailed results were visual saliency figure.
